# Machine learning-based prediction and mechanistic insight into PFAS adsorption on carbon-based materials

**DOI:** 10.1039/d5ra07898a

**Published:** 2025-12-08

**Authors:** Yanliang Lu, Fangfang Ding, Guchun Wang, Yabin Li, Zhitao Guo, Peiyao Pang, Baojun Wang, Jue Liu

**Affiliations:** a National & Local Joint Engineering Research Center of Metrology Instrument and System, College of Quality and Technical Supervision, Hebei University Baoding 071002 China wbj498@163.com; b Key Laboratory of Tropical Island Land Surface Processes and Environmental Changes of Hainan Province, School of Geography and Environmental Sciences, Hainan Normal University Haikou 571158 China jueliu@buaa.edu.cn

## Abstract

Carbon-based materials hold significant potential in environmental remediation, as they can effectively remove per- and polyfluoroalkyl substances (PFAS) through adsorption, thereby influencing their environmental behavior and associated risks. However, due to the complexity of the physicochemical properties of carbon-based materials, the molecular diversity of PFAS—including variations in chain length, functional groups, and degree of fluorination—as well as differences in environmental conditions, it remains challenging to fully elucidate the adsorption mechanisms solely through experimental approaches. In this study, a gradient boosting decision tree (GBDT) model was developed and optimized to systematically predict the adsorption performance of carbon-based materials toward PFAS. The GBDT model demonstrated excellent predictive accuracy on the test dataset, achieving an *R*^2^ of 0.96 and a root mean square error (RMSE) of 0.02. Model interpretation using Shapley additive explanations (SHAP) and partial dependence plots revealed that environmental conditions contributed the most to adsorption, followed by the physicochemical characteristics of carbon-based materials and the molecular features of PFAS. Specifically, solution pH, the number of fluorine atoms within PFAS molecules, temperature, and the pore structure of carbon-based materials were identified as the most influential factors, with electrostatic interactions and hydrophobic–hydrophilic character are likely the dominant mechanisms. This study provides a novel perspective that integrates machine learning with environmental chemistry to enhance understanding of PFAS–carbon interactions, offering valuable insights for environmental risk assessment and the rational design of functional materials.

## Abstract 2

Machine learning reveals the dominant factors controlling PFAS adsorption on carbon-based materials, highlighting the role of hydrophobic and electrostatic interactions.

## Graphical abstract

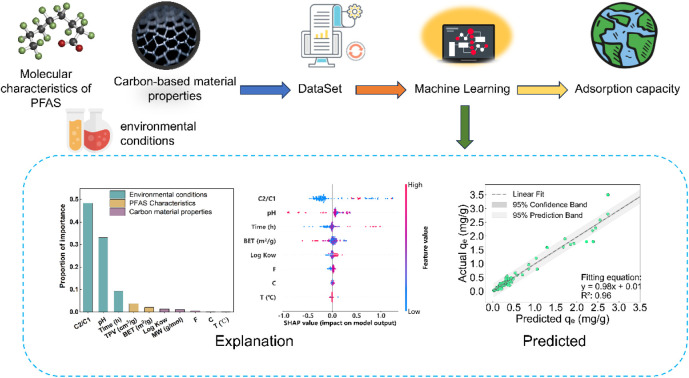

## 1. Introduction

Per- and polyfluoroalkyl substances (PFAS) are an emerging class of contaminants whose potential hazards have not yet received sufficient attention. Due to their unique chemical properties, PFAS are virtually non-degradable once released into the environment, earning them the moniker “forever chemicals” from the American Chemical Society. Currently, perfluorooctanoic acid (PFOA) and perfluorooctane sulfonate (PFOS) are recognized as emerging pollutants. Studies indicate that in 66 cities across China, over 40% of drinking water sources contain PFOA and PFOS concentrations exceeding the California notification level of 5.1 ng L^−1^ for PFOA and 6.5 ng L^−1^ for PFOS, potentially affecting approximately 192.6 million residents.^1^ The exceptional stability of the carbon–fluorine (C–F) bond, one of the strongest single bonds in nature, renders PFAS highly resistant to chemical and biological degradation.^2^ This molecular stability imparts PFAS with remarkable thermal resistance, chemical inertness, and long-term environmental persistence, which underpins their widespread industrial applications. These include surface coatings, protective materials, food packaging, firefighting foams, metal plating, and electronic manufacturing. PFAS are also extensively used in the textile industry for producing water-, stain-, and oil-repellent fabrics and garments, such as outdoor apparel, waterproof carpets, sofas, and curtains.^3^ Owing to their ubiquitous use in daily life,^4^ PFAS can enter ecosystems through multiple pathways and undergo environmental dissemination. Their risks to human health and ecological systems primarily arise from their persistence, bioaccumulative potential, and inherent toxicity.^5^

Current strategies for addressing PFAS contamination include advanced oxidation, membrane separation, ion exchange, and adsorption.^6^ Among these, adsorption has attracted particular attention in practical applications due to its low energy consumption, capability for on-site remediation, and lack of harmful byproducts. Carbon-based materials, such as activated carbon, graphene, biochar, and carbon nanotubes, exhibit excellent PFAS removal performance owing to their abundant porous structures and tunable surface functional groups, making them a central focus of PFAS adsorption research.^7^ Given that the adsorption behaviors and mechanisms can vary significantly depending on the type of carbon material, the specific PFAS species, and the complex environmental conditions, relying solely on experimental studies for systematic investigation is often time-consuming and resource-intensive. Therefore, achieving rapid prediction of PFAS adsorption performance on carbon-based materials using pre-existing datasets has become a critical challenge for improving environmental risk assessment efficiency and guiding the design and optimization of adsorbent materials. However, traditional methods still face limitations in delivering high-precision predictions.

In recent years, with the rapid development of data science and artificial intelligence, machine learning (ML) has gradually emerged as a powerful tool in environmental remediation and functional materials research.^8^ By deeply mining experimental and literature-derived datasets, ML models can capture underlying relationships among variables, establish predictive frameworks for adsorption performance, and identify key factors that critically influence the adsorption process.^9^ Compared with traditional statistical approaches, machine learning offers superior predictive accuracy and is capable of revealing complex nonlinear effects and interactions among features, providing new methodological insights into adsorption mechanisms. Although some studies have attempted to use machine learning to predict pollutant adsorption, systematic investigations specifically targeting the interactions between carbon-based materials and PFAS remain relatively limited. Recent ML-based adsorption studies have mainly targeted pollutants such as ammonia nitrogen, dyes, and methylene blue, leaving PFAS largely unexplored.^56–59^ Bibliometric analyses have also highlighted the absence of ML-driven adsorption prediction frameworks for PFAS.^60^ However, ML studies focusing on PFAS adsorption on carbon-based materials remain scarce. Therefore, a dedicated and comparative ML evaluation framework is needed to address this gap and improve the understanding of PFAS–material interactions.

A systematic investigation was conducted to examine the adsorption behavior of PFAS by carbon-based materials, highlighting their potential for environmental remediation.^10–14^ To comprehensively understand the factors influencing the adsorption process, a variable system was constructed from three perspectives: environmental conditions (solution pH, reaction time, initial concentration ratio), physicochemical properties of carbon-based materials (specific surface area, pore volume), and intrinsic properties of PFAS (carbon chain length, number of fluorine atoms, logarithm of the octanol–water partition coefficient, log *K*_ow_). Multiple modeling approaches were employed to predict the adsorption performance of PFAS on carbon-based materials based on these variables. By evaluating the performance of 13 machine learning models—including traditional regression, ensemble learning, and deep learning—this study, combined with feature importance analysis, identified the relative contributions of different factors to adsorption behavior. Comparative analysis of model predictions further clarified their applicability and relative advantages in the carbon-based material–PFAS adsorption system.

## 2. Methodology

**Fig. 1:**
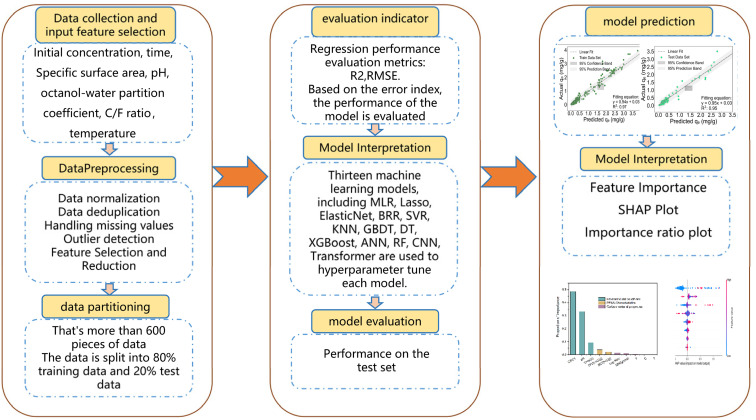
Workflow of models for evaluating PFAS adsorption on carbon-based materials.

Fig. 1 illustrates the overall research framework, which encompasses data acquisition, visualization and preprocessing, model construction and validation, as well as result interpretation based on the GBDT method. This framework is designed to predict the adsorption performance of carbon-based materials toward PFAS and to identify the key factors governing adsorption behavior. These factors involve the structural and physicochemical properties of carbon-based materials, the molecular descriptors of PFAS, and the influence of external environmental conditions.

### 2.1 Data collection and preprocessing of PFAS adsorption on carbon-based materials

High-quality input data are essential for model training, as invalid or anomalous data may cause overfitting and subsequently lead to inaccurate predictions. Therefore, data collection and screening were carried out rigorously to ensure the robustness of the model. Publications from the past two decades were retrieved from the Web of Science database using multiple keyword combinations. The primary keywords used in this study included adsorbent (*e.g.*, biochar, activated carbon), adsorbate (*i.e.*, per- and polyfluoroalkyl substances, PFAS), and interaction process (*i.e.*, adsorption). To avoid the negative influence of irrelevant or inconsistent information, studies with low data relevance, abnormal results, or lacking explicit adsorption capacity values were excluded.

Ultimately, 37 publications were selected, from which a total of 605 valid data entries were extracted (for details on the included references, see Text S5). All adsorption data used in this study were derived from experimental results reported in the original literature. In some cases, adsorption capacity data were directly provided in tabulated form, while in others, data were presented graphically as adsorption kinetics or isotherms. For the latter, adsorption data were digitized using the Screen Reader Tool of Origin 2021 Pro.

After preprocessing, 10 key parameters were extracted for subsequent model training: initial adsorbent concentration (mg L^−1^), PFAS concentration (mg L^−1^), surface area of the carbon-based material (m^2^ g^−1^), total pore volume of the carbon material, solution pH, PFAS carbon chain length (C), number of fluorine atoms (F), molecular weight, reaction temperature (°C), and adsorption capacity (mg g^−1^).

### 2.2 Data structure exploration and preprocessing strategy

After the raw data were collected, an initial visualization and distribution analysis of the dataset were performed. Obvious outliers (extremely large or small data points) were manually removed to improve data quality and reduce noise interference. To ensure proper model training, missing values were imputed using the SimpleImputer module from scikit-learn.^15^ We additionally evaluated multivariate imputation with IterativeImputer, but the SimpleImputer-based scheme exhibited slightly better generalization performance and was therefore retained in the final models. In this study, column-wise mean imputation was applied, whereby missing values in each feature were replaced with the mean of the non-missing values in the corresponding column. Secondly, to address the potential multicollinearity between carbon chain length and fluorine content, principal component analysis (PCA) was applied to perform dimensionality reduction.^16,17^ The first principal component was extracted to replace the original C and F descriptors, thereby retaining the essential information while minimizing redundancy. Subsequently, all feature variables were standardized using *z*-score normalization (mean = 0, standard deviation = 1) to eliminate unit discrepancies and enhance comparability across features.^18^ In the modeling phase, the preprocessed dataset was randomly partitioned into two subsets, with 20% reserved as the testing dataset and the remaining 80% as the training dataset, while fixing the random seed to ensure reproducibility of the results.

To assess the linear relationship between each input feature and the target variable (adsorption capacity), the Pearson Correlation Coefficient (PCC) was introduced as an initial statistical tool, following common practices in adsorption and materials studies.^19,20^ PCC is one of the most commonly used measures of correlation, quantifying both the strength and direction of a linear relationship between two continuous variables. By calculating the PCC between each feature and the target, dominant variables, potential multicollinearity, and abnormal features can be identified before modeling, providing a basis for subsequent feature selection and model construction. Redundant input variables can be removed to reduce computational cost, mitigate overfitting, and improve model compatibility and predictive accuracy. The PCC is mathematically defined as follows, eqn (1):^21^1
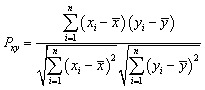
In the PCC formula, *P*_*xy*_ corresponds to the Pearson correlation coefficient, while *x*_*i*_ and *y*_*i*_ represent the true values of the two input features for the *i*-th data point. *n* denotes the total number of data points, and *x̄* and *ȳ* are the mean values of the two input features, *X* and *Y*, respectively. The value of *P*_*xy*_ ranges from −1 to 1, where 0 indicates no correlation, and 1 or −1 represents strong positive or negative correlation, respectively. Finally, an eight-dimensional matrix of normalized input features and a corresponding vector of the normalized output were obtained for model training, testing, and validation.

To evaluate the nonlinear associations between the input and target variables, Spearman's rank correlation coefficient and Kendall's rank correlation coefficient were employed, with their mathematical formulations presented in eqn (2):^22^2
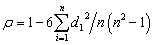
Here, *d*_*i*_ represents the difference in ranks between the two variables for the *i* observation, and *n* denotes the total number of samples.3
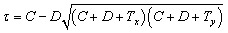
In this context, *C* counts all concordant pairs where (*x*_*i*_ − *x*_*j*_)(*y*_*i*_ − *y*_*j*_) > 0, and *D* counts discordant pairs where (*x*_*i*_ − *x*_*j*_)(*y*_*i*_ − *y*_*j*_) < 0. *T*_*x*_ and *T*_*y*_ indicate the number of ties in *x* and *y*, respectively.

### 2.3 Model development and evaluation

In this study, multiple machine learning algorithms were employed to develop predictive models and conduct a comparative performance analysis. The selected algorithms included Gradient Boosting Decision Trees (GBDT), Extreme Gradient Boosting (XGBoost), Multiple Linear Regression (MLR), Bayesian Ridge Regression (BRR), Random Forests (RF), Artificial Neural Networks (ANN), Support Vector Regression (SVR), Elastic Net Regression (ElasticNet), k-Nearest Neighbors (KNN), Decision Trees (DT), Lasso Regression, Convolutional Neural Networks (CNN), and Transformer models. These algorithms were implemented to predict the target output variable, *i.e.*, the adsorption capacity of PFASs on carbon-based materials. To ensure optimal predictive performance, hyperparameter optimization was carried out using a grid search approach (Text S1). All model computations were performed in PyCharm 2020.1.5 (Python 3.9), leveraging appropriate machine learning libraries.

For model evaluation, the root mean square error (RMSE) and the coefficient of determination (*R*^2^) were employed as the primary performance metrics. RMSE reflects the average deviation between the predicted and actual values, serving as one of the key indicators for assessing regression error.^23^ In contrast, *R*^2^ quantifies the extent to which the model can explain the variance of the target variable. Ideally, a smaller RMSE combined with an *R*^2^ value approaching 1 indicates higher predictive accuracy. The mathematical formulations of these two metrics are given as follows:4
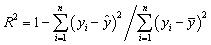
5
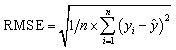
In the above equations, *y*_*i*_ denotes the observed (true) value, *ŷ* represents the predicted value, *ȳ* is the mean of the observed samples, and *n* refers to the total number of data points in the dataset.

### 2.4 GBDT model interpretation

The feature importance of the Gradient Boosting Decision Tree (GBDT) model was quantified using the model feature importances attribute from the Scikit-learn library. This approach evaluates the relative importance of each feature based on its average contribution to node purity (*e.g.*, Gini index or entropy) during the decision-making process, thereby providing an initial interpretation of the model's decision mechanism.^24^ It is worth noting that the GBDT model is relatively sensitive to noisy features and hyperparameter settings, particularly when the learning rate (learning_rate) is large, the number of boosting iterations (n_estimators) is high, or the tree depth (max_depth) is unrestricted.^25^ To ensure stability and avoid overfitting, model hyperparameters were tuned within defined ranges (learning_rate: 0.05–0.40; n_estimators: 30–300; max_depth: 2–15), and model evaluation was conducted on a fixed 80/20 train–test split using *R*^2^ and RMSE as the performance metrics.

The SHAP method was introduced to enhance model interpretability while providing a unified framework to quantify the marginal contribution of each input feature in individual predictions.^26^ This approach brings transparency to complex machine learning models such as GBDT, thereby facilitating the understanding of their decision-making mechanisms. The theoretical foundation of SHAP is detailed in SI Text S2.

Furthermore, to explore potential nonlinear relationships and interaction effects between input variables and the target output, Partial Dependence Plot (PDP) was employed, following similar practices in recent adsorption studies.^27,28^ Also grounded in Shapley value theory, this approach reveals the response trends of model predictions to changes in individual features. The computation and visualization of SHAP values and partial dependence plots were conducted using the SHAP module in combination with the Scikit-learn toolkit, with detailed programming procedures and parameter settings provided in SI Text S3.

## 3. Results and discussion

### 3.1 Descriptive statistics

**Fig. 2:**
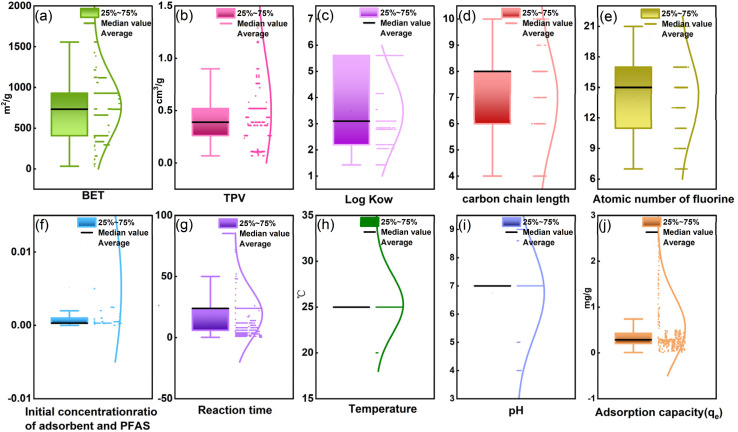
Box diagrams and data distributions of each feature. The abbreviated features with their respective units are as follows: (a) specific surface area BET (m^2^ g^−1^); (b) total pore volume TPV (cm^3^ g^−1^); (c) log *K*_ow_; (d) carbon chain length; (e) atomic number of fluorine; (f) initial concentration ratio of adsorbent to PFAS; (g) reaction time (h); (h) temperature (°C); (i) solution pH; (j) adsorption capacity *q*_e_ (mg g^−1^).

Ten features were selected as input variables based on the physicochemical properties of the carbon materials, the chemical characteristics of PFAS, and environmental conditions (Fig. 2). Among these, the specific surface area and total pore volume of the adsorbents were considered. In the dataset, the specific surface area ranged primarily from 33.6 to 2341 m^2^ g^−1^ (Fig. 2a). Generally, the magnitude of the specific surface area determines the number of adsorption sites, with larger surface areas corresponding to improved adsorption performance. The total pore volume of the carbon materials was mainly concentrated within the range of 0.07–3.79 cm^3^ g^−1^ (Fig. 2b). The pore volume dictates the internal space available for accommodating molecules. Larger total pore volumes provide more space for PFAS molecules to enter and reside within the material, thereby enhancing adsorption capacity.^29^

The dataset incorporated several molecular descriptors of PFAS, including log *K*_ow_, carbon chain length, number of fluorine atoms, and molecular weight. The log *K*_ow_ values in the dataset ranged from 1.43 to 5.61 (Fig. 2c), which are typically used to characterize the balance between hydrophilicity and hydrophobicity of compounds. Hydrophobic–hydrophilic interactions play a critical role in the adsorption behavior of PFAS.^30^ Due to their fluorocarbon chains, PFAS molecules exhibit strong hydrophobicity, which promotes hydrophobic–hydrophobic interactions with the hydrophobic domains of carbon-based materials; this mechanism is recognized as one of the dominant pathways driving PFAS adsorption. As shown in Fig. 2d, the carbon chain length ranged from 4 to 10. Longer-chain PFAS, with stronger hydrophobicity, are generally more readily adsorbed by carbon materials compared to short-chain PFAS.^31^ Moreover, tailoring the surface hydrophobicity of carbon adsorbents (*e.g.*, *via* hydrophobic modification) can optimize adsorption performance toward different PFAS types. As shown in Fig. 2e, the number of fluorine atoms in PFAS ranges from 7 to 21, and PFAS molecules with different fluorine counts exhibit distinct hydrophobicity–hydrophilicity characteristics.^32^

In adsorption experiments, four environmental factors were considered: the initial solution concentration ratio of carbon to PFAS, contact time, temperature, and solution pH. The concentration ratio (*C*_1_/*C*_2_, both expressed in mg ^−1^) is a dimensionless parameter representing the relative concentration of the carbon adsorbent (*C*_1_) to the PFAS solute (*C*_2_). As shown in Fig. 2f, this ratio affects adsorption efficiency by determining the relative number of available adsorption sites per PFAS molecule. In the dataset, the contact time ranged from 0.25 to 238 h (Fig. 2g); generally, adsorption capacity increases with time until equilibrium is reached. The temperature varied between 20 and 40 °C (Fig. 2h). Since the adsorption of perfluorooctanesulfonic acid (PFOS) is endothermic,^55^ temperature positively affects both the adsorption kinetics and overall adsorption capacity, indicating that energy input facilitates the adsorption process. In the adsorption experiments, the pH range varied as shown in Fig. 2i, and pH was found to have a significant effect on adsorption efficiency. Generally, pH influences adsorption by modulating the hydrophilicity–hydrophobicity balance of PFAS molecules and by altering electrostatic interactions between PFAS and the carbon surface. From a machine learning and chemistry perspective, pH can be treated as a critical input feature that impacts PFAS–adsorbent interactions, thereby affecting the predictive modeling of adsorption performance.

Due to severe collinearity between the carbon chain length (C) and the number of fluorine atoms (F), directly inputting these as independent features could lead to multicollinearity, affecting model stability and interpretability. To address this, principal component analysis (PCA) was applied to reduce the dimensionality of these correlated features. PCA transforms the original correlated variables into a set of new orthogonal variables through linear combinations, preserving as much variance from the original data as possible. Using this approach, C and F were integrated into a single composite variable, named CF-PCA, that maintains a high correlation with the original features and effectively represents the combined characteristics of PFAS chain length and fluorine content. In subsequent model development and analysis, using this composite variable instead of the original C and F not only mitigates multicollinearity but also has negligible impact on predictive performance. For the theoretical basis and implementation of PCA, please refer to SI Text S4.

### 3.2 Pearson correlation coefficient (PCC)

The Pearson correlation coefficient (PCC) is a widely used measure of linear correlation in multivariate statistical analysis, which quantitatively assesses the degree of linear dependence between continuous variables under approximately normal distributions.^34^ In this study, PCC analysis was first conducted to examine the linear relationships among all input features. The results indicate that the physical properties of carbon materials (such as specific surface area, BET, and total pore volume, TPV) are strongly positively correlated with the molecular weight (MW), octanol–water partition coefficient (log *K*_ow_), and CF-PCA of PFAS molecules, with PCC values of 0.8, 0.84, and 0.92, respectively (Fig. S1). This high correlation suggests a strong interrelationship between BET and TPV, as well as inherent associations between MW, log *K*_ow_, and CF-PCA arising from the intrinsic properties of PFAS molecules. The analysis indicates a certain degree of collinearity and information redundancy among these variables. To reduce the impact of redundant features on model stability and interpretability, the total pore volume of the carbon materials and the molecular weight of PFAS were excluded in subsequent modeling, thereby mitigating multicollinearity and enhancing the model's generalization capability. It should be noted that PCC alone was not used to directly identify abnormal features; it served as a preliminary linear-screening tool. PCC is valid when variables are continuous and approximately linearly related. Features flagged by high or low PCC values were further evaluated using Spearman and Kendall rank correlations to detect nonlinear associations, and variance inflation factor (VIF) analysis to assess multicollinearity. Apparent anomalies were also cross-checked against the original datasets and domain knowledge before removal or transformation, ensuring that feature screening is statistically and physically interpretable.^63^

To further investigate whether potential nonlinear associations exist among the feature variables, this study employed the Spearman rank correlation coefficient and the Kendall rank correlation coefficient as evaluation tools. These two non-parametric statistical approaches are widely applied in assessing nonlinear dependencies between variables.^35^ The analysis results indicate that, except for the relationship between CF-PCA and log *K*_ow_, the correlation coefficients of all retained features fall within the range of −0.5 to 0.5 (Fig. S2 and S3), suggesting no significant evidence of nonlinear coupling effects.

**Fig. 3:**
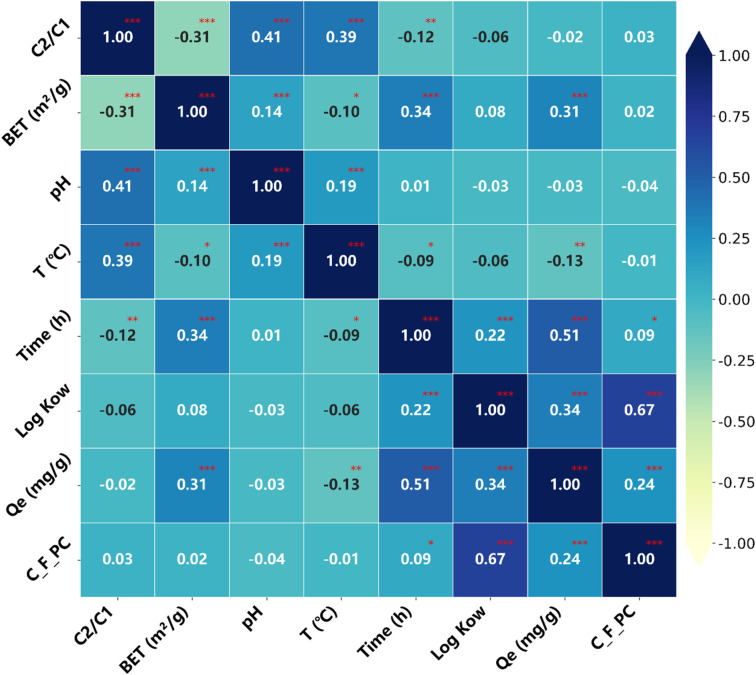
Pearson correlation matrix of the selected features and the adsorption capacity. The color scale is fixed between −1 and 1, where the color intensity represents the strength and sign of the linear correlation (−1: strong negative, +1: strong positive, 0: weak or no correlation). The numbers in each cell denote the Pearson correlation coefficients (*r*), while the star symbols indicate the statistical significance levels based on the corresponding *p*-values (*p < 0.05, **p < 0.01, ***p < 0.001).

Under the Pearson correlation coefficient (PCC) analysis, the environmental factors (such as the initial concentration ratio of carbon to PFAS, pH, time, and temperature) and the intrinsic physicochemical properties of PFAS exhibited good independence, indicating their suitability as important individual contributors for model prediction. Among these, the PCC between CF-PCA and log *K*_ow_ was 0.67 (Fig. 3). Moreover, as shown in Fig. S2 and S3, the Spearman and Kendall rank correlation coefficients between CF-PCA and log *K*_ow_ were 0.86 and 0.69, respectively. Although all of the correlation metrics exceeded 0.5, suggesting a certain degree of association, further diagnosis using the variance inflation factor (VIF) revealed values below 2 (Fig. S4), which are far lower than the commonly used conservative threshold for indicating potentially problematic multicollinearity (VIF = 5).^61,62^ Therefore, the correlation between CF-PCA and log *K*_ow_ was insufficient to adversely affect model performance. Retaining the log *K*_ow_ feature is thus appropriate, as it provides valuable molecular hydrophobicity information without introducing multicollinearity issues.

For the remaining features, PCC values were within the range of −0.5 to 0.5 (Fig. 3), indicating no significant linear correlations. To further ensure that multicollinearity would not interfere with model training and interpretability, VIF was systematically applied as a diagnostic tool. As illustrated in Fig. 6, all input variables showed VIF values ranging from 1.18 to 1.89, confirming that feature intercorrelations were weak. Consequently, in the machine learning framework, each feature can be interpreted as making an independent contribution to PFAS adsorption prediction, without compromising model performance or interpretability.

### 3.3 Model development and evaluation

**Fig. 4:**
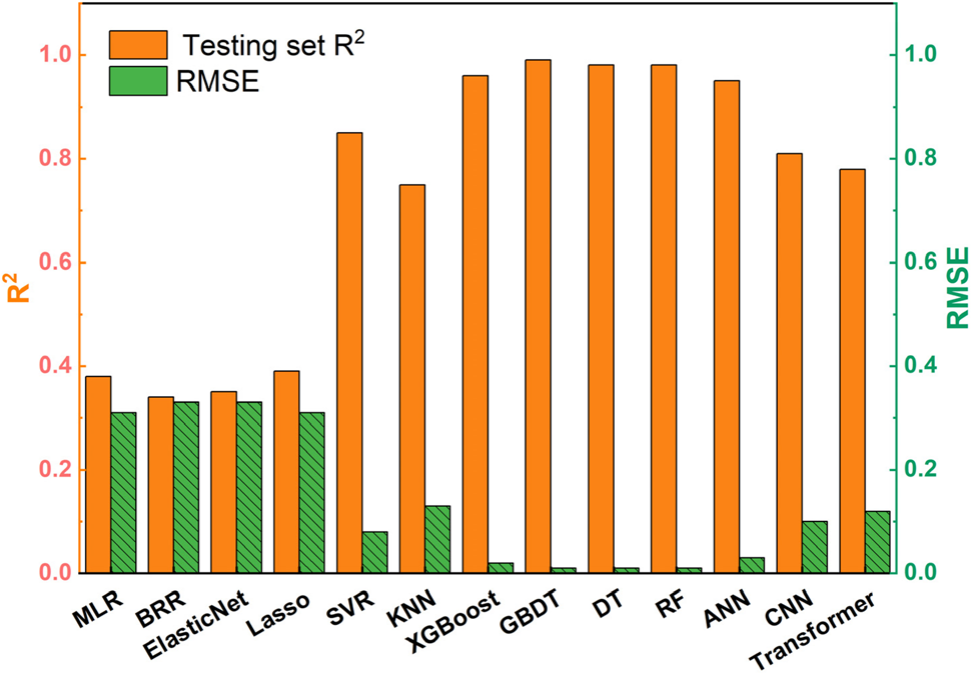
Evaluation of different model performances on the testing dataset.

In this study, a systematic comparison of various regression and machine learning models was conducted to predict the adsorption behavior of PFAS on carbon-based materials, based on optimized training procedures. The models included traditional regression approaches, such as MLR, BRR, ElasticNet, and Lasso, as well as widely used machine learning algorithms, including SVR, ANN, KNN, XGBoost, GBDT, DT, and RF. Additionally, deep learning models, including CNN, and Transformer architectures, were applied Model performance was evaluated using root mean square error (RMSE) and the coefficient of determination (*R*^2^) (see Table 1 and Fig. 4). Lower RMSE values and *R*^2^ values closer to 1 indicate better model fitting and higher predictive accuracy.^36^

**Table 1:** Comparative evaluation of ML model performances of different data sets

Model	Training set *R*^2^	Testing set *R*^2^	Training set RMSE	Testing set RMSE	Remarks
MLR	0.38	0.30	0.31	0.35	Low accuracy
BRR	0.34	0.30	0.33	0.36	Low accuracy
ElasticNet	0.35	0.28	0.33	0.37	Low accuracy
Lasso	0.39	0.27	0.31	0.37	Low accuracy
SVR	0.85	0.33	0.08	0.32	Overfits
KNN	0.75	0.52	0.13	0.19	Moderate
XGBoost	0.96	0.96	0.02	0.02	High accuracy
GBDT	0.99	0.96	0.01	0.02	Best overall
DT	0.98	0.84	0.01	0.07	Slight overfitting
RF	0.98	0.87	0.01	0.06	Slight overfitting
ANN	0.95	0.87	0.03	0.06	Moderate
CNN	0.81	0.68	0.10	0.16	Underfits
Transformer	0.78	0.6	0.12	0.11	Underfits

Experimental results indicate that traditional linear regression models generally underperform in predicting PFAS adsorption. For instance, the MLR model achieved only *R*^2^ = 0.30 and RMSE = 0.35 on the testing, demonstrating its limited capacity to capture the complex linear and nonlinear interactions between input features and the response variable. Although BRR is theoretically suitable for small-sample modeling, its performance on the testing set was also suboptimal, with *R*^2^ = 0.30 and RMSE = 0.36, likely due to limitations in prior specification. The ElasticNet model, which in principle balances variable selection and multicollinearity handling, struggled to reconcile sparsity and stability when strong and weak features coexisted, resulting in testing *R*^2^ = 0.28 and RMSE = 0.37. Similarly, the Lasso model exhibited unsatisfactory testing performance (*R*^2^ = 0.27, RMSE = 0.37), presumably constrained by its inherent linear regression framework, which limits its ability to fully capture potential nonlinear relationships among input variables.

Different types of machine learning (ML) models exhibited significant variations in predictive performance. Among the tested models, the SVR model exhibited suboptimal performance in this study. The *R*^2^ values on the training and testing sets were 0.85 and 0.33, respectively, indicating that while the model performed well on the training set, its predictive accuracy declined sharply on the testing set (*R*^2^ = 0.33). Moreover, many data points fell outside the 95% confidence interval in the testing phase, suggesting that the SVR model suffered from poor generalization ability. This performance decline is typically attributed to overfitting, and the relatively small size of the training dataset further constrained the model. The limitations of SVR are likely associated with challenges in kernel selection and hyperparameter tuning on small-scale datasets, which hinder its ability to effectively capture nonlinear structures in complex data.^37^

The KNN algorithm demonstrated moderate predictive performance in this study. The KNN model achieved *R*^2^ values of 0.75 and 0.53 for the training and testing sets, respectively, indicating good fit on the training data but a noticeable decline in predictive accuracy on the test set. This performance drop is primarily attributed to KNN's strong reliance on the local distribution of samples: regions of high data density in the training set enable accurate fitting, whereas test samples located in sparse regions are prone to larger prediction errors.^38^ Additionally, this behavior may result from imbalanced data distributions and the model's limited generalization capability for unseen samples. In contrast, the RF model exhibited *R*^2^ values of 0.98 and 0.87 for the training and testing sets, respectively, reflecting its strong fitting capacity on training data. However, the relatively lower performance observed on the test set indicates that the model's generalization may be constrained by variations in data distribution, potential overfitting, and sensitivity to noise, suggesting that the predictive robustness of the random forest model still faces certain limitations.^39^

The artificial neural network (ANN) model achieved *R*^2^ values of 0.95 and 0.87 on the training and testing sets, respectively. Although ANN possesses a certain capability for nonlinear modeling, its performance is strongly dependent on parameter optimization and sample size. As a result, its learning ability is limited under small- to medium-sized datasets, preventing the model from fully realizing its potential. In comparison, the DT model yielded an *R*^2^ of 0.84 on the testing, demonstrating a certain level of predictive capability. However, due to its high sensitivity to data fluctuations, DT tends to overfit local features of the training set, thereby constraining its generalization ability and reproducibility.^40^

**Fig. 5:**
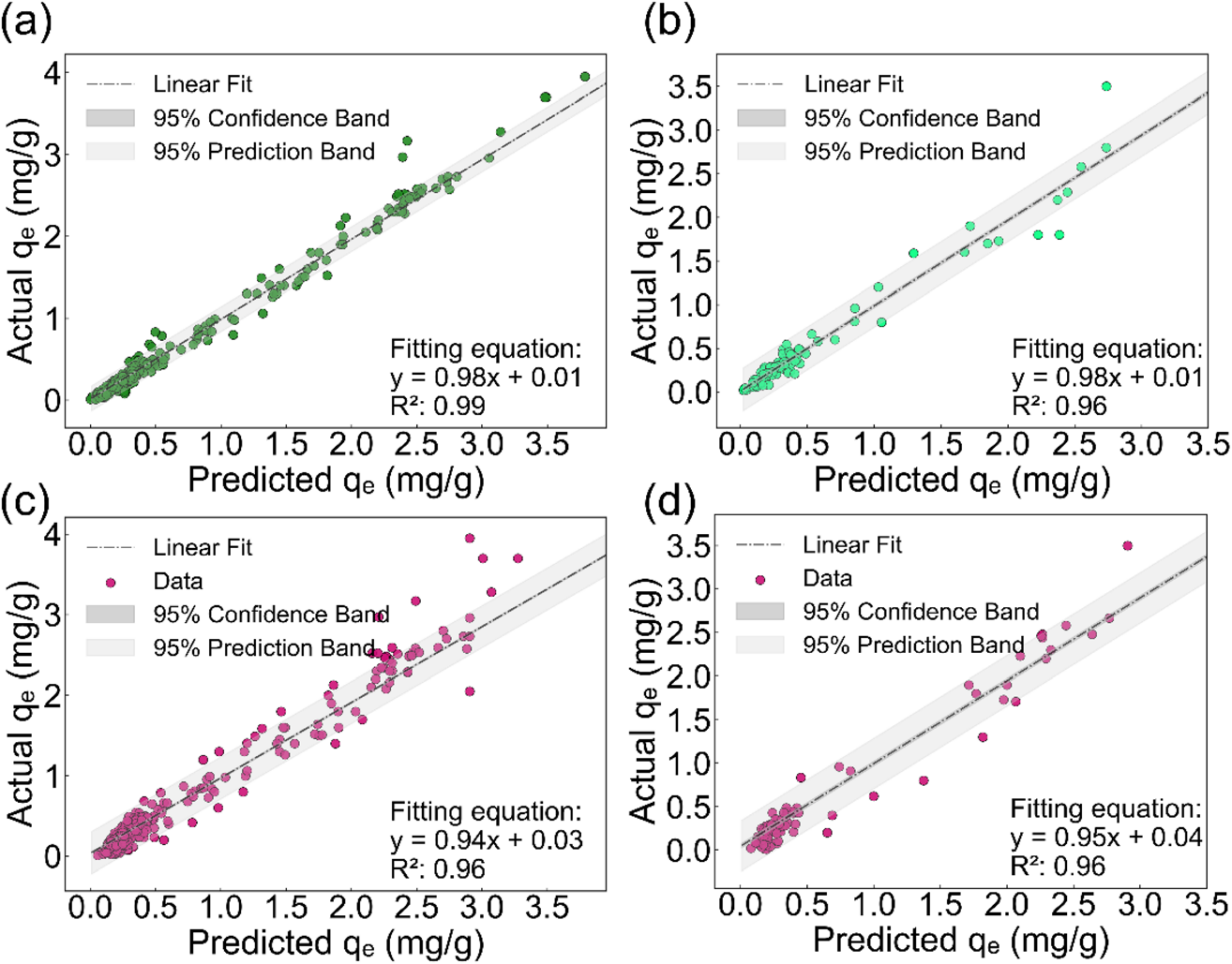
Results of GBDT (a and b) and XGBoost (c and d) for predicting PFAS adsorption on carbon-based materials. Panels (a) and (c) show the results for the training set, while panels (b) and (d) show the results for the test set.

It is noteworthy that the ensemble learning models XGBoost and GBDT exhibited the best performance among all the machine learning algorithms. Both models achieved an *R*^2^ as high as 0.96 on the testing set, with an RMSE of only 0.02. Moreover, the vast majority of the predicted values fell within the 95% confidence interval (Fig. 5). XGBoost leverages the gradient boosting framework combined with regularization strategies to achieve high-precision predictions; however, its model architecture is complex, hyperparameter tuning is cumbersome, and it requires substantial computational resources. In contrast, the GBDT model maintains excellent predictive performance while incurring relatively low computational cost.

To evaluate whether the GBDT model exhibited overfitting, key hyperparameters of the model were systematically tuned and a sensitivity analysis was conducted. As shown in Fig. 8, the *R*^2^ values of both the training and testing sets fluctuated only slightly across different hyperparameter combinations, and no significant divergence between the two was observed. This indicates that the GBDT model developed in this study demonstrates strong generalization ability without evident overfitting.

This study also explored the application of deep learning methods for predicting PFAS adsorption by carbon-based materials; however, the overall performance was suboptimal, with test set *R*^2^ values of 0.68 for CNN and 0.60 for Transformer. Specifically, the CNN relies on convolutional layers to extract local features and is more suited for grid-structured data, limiting its effectiveness when handling non-grid inputs. In contrast, the Transformer model leverages a self-attention mechanism to capture long-range dependencies, but its modeling advantages typically require large-scale datasets, making its performance constrained under small- to medium-sized sample conditions.

### 3.4 Correlation analysis

**Fig. 6:**
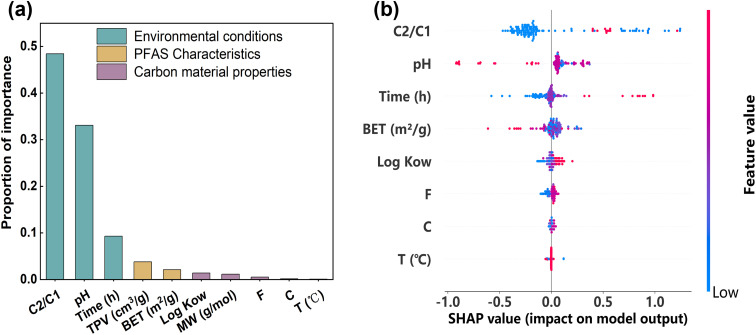
Feature importance (a) and SHAP values (b) of input variables on carbon adsorption of PFAS. The color bar on the right side represents the value of the input variable.

Given the superior predictive performance of the GBDT model for PFAS adsorption by carbon-based materials, this study further leveraged the model to quantify the relative importance of each input variable during the adsorption process. The analysis aimed to evaluate the contribution of individual features to the overall adsorption behavior. The results indicate that all input features—including the physicochemical properties of carbon materials, molecular attributes of PFAS, and environment-related factors—exert varying degrees of influence on adsorption. Among these factors, environmental conditions exerted the most significant driving effect on adsorption behavior, followed by the physicochemical properties of carbon-based materials, while the chemical characteristics of PFAS contributed comparatively less to the model predictions (Fig. 6a).

**Fig. 7:**
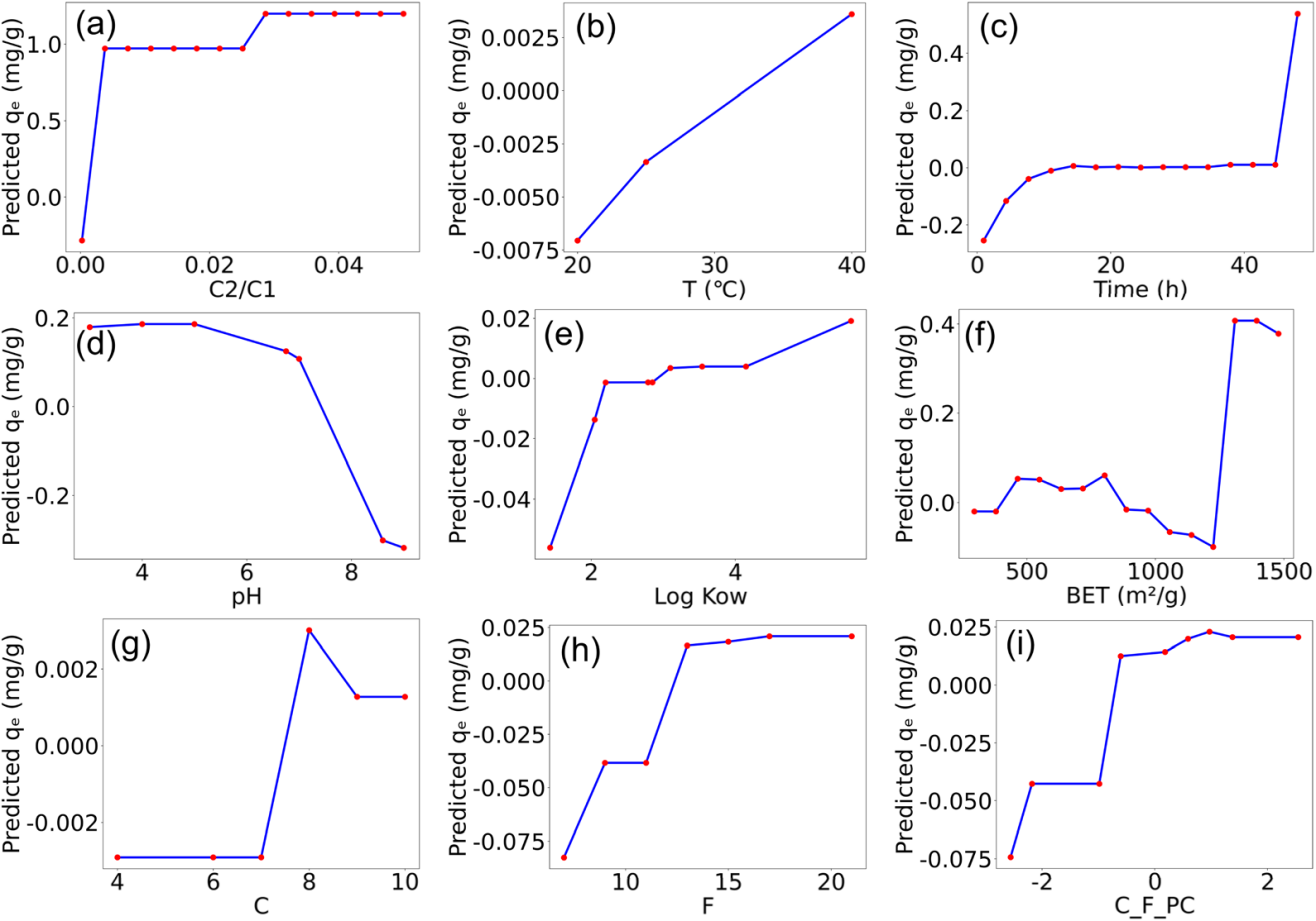
Partial dependence plots of the selected features on the predicted adsorption capacity, including initial concentration ratio of adsorbent and PFAS (a), temperature (°C) (b), reaction time (h) (c), pH (d), log *K*_ow_ (e), BET (m^2^ g^−1^) (f), carbon chain length (g), atomic number of fluorine (h), and C_F_PC (i). The *x*-axes show the feature values with their corresponding physical units, and the *y*-axis denotes the predicted *q*_e_ (mg g^−1^). Each partial dependence curve is evaluated at a set of grid points, which are explicitly shown as red markers along the curves. C_F_PC represents the first principal component derived from the standardized C and F variables.

In adsorption experiments, multiple factors influence the adsorption capacity. In the following, we analyze the effect of each factor considered in this study on adsorption performance. Among the environmental factors, the initial concentration ratio of carbon to PFAS was identified as the most important input variable. The SHAP plot (Fig. 6b) shows that most data points have positive SHAP values, indicating a favorable effect on adsorption. As shown in the partial dependence plot (Fig. 7a), PFAS adsorption by carbon increases with the initial concentration ratio of carbon to PFAS. However, a higher carbon-to-PFAS ratio does not necessarily result in greater adsorption efficiency, because once the ratio reaches a threshold, the adsorption sites on carbon corresponding to PFAS are fully occupied, and additional carbon does not enhance adsorption. Over time, the majority of adsorption sites on the carbon surface become occupied, which limits the adsorption rate of PFAS molecule.^41,42^ According to adsorption kinetics studies, when the PFAS concentration remains constant, increasing the carbon concentration leads to higher adsorption efficiency until saturation is reached.

**Fig. 8:**
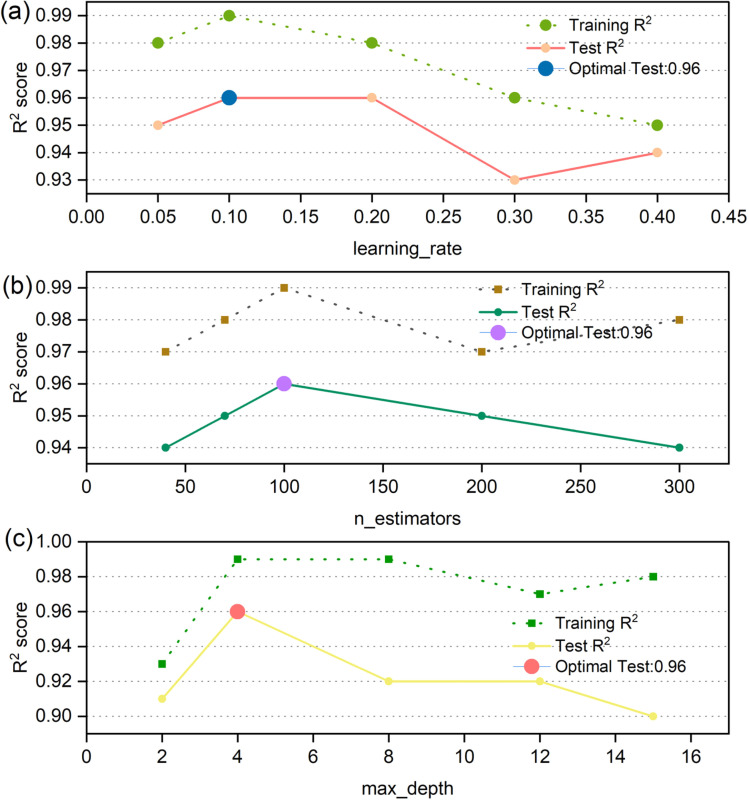
*R* ^2^ score from dynamically adjusting the three parameters of GBDT: learning_rate (a), n_estimators (b), and max_depth (c).

Temperature plays a significant role in the adsorption of PFAS by carbon materials. Most experiments were conducted at or slightly above or below room temperature, so the observed temperature range is relatively narrow. As shown in the partial dependence plot (Fig. 7b), within the 20–40 °C range, the adsorption capacity increases with rising temperature. This indicates that temperature positively influences both the adsorption kinetics and capacity of PFAS, primarily because the adsorption process of PFAS is endothermic.^33^ At higher temperatures, enhanced adsorption capacity may result from accelerated intraparticle diffusion of adsorbates into the pores of the carbon adsorbent,^43^ facilitating PFAS uptake. Therefore, within an appropriate temperature range, increasing temperature can promote the adsorption of PFAS by carbon materials.^44^

The temporal factor plays a critical role in the adsorption capacity during the PFAS adsorption process. From the partial dependence plot (Fig. 7c), it is evident that the adsorption capacity gradually approaches saturation within the initial 0–45 h. This trend can be attributed to the abundance of available adsorption sites on the activated carbon surface at the early stage, where PFAS molecules rapidly occupy the surface as well as the macropore/mesopore adsorption sites, which facilitates PFAS uptake.^41^ However, as adsorption progresses, the number of accessible sites on the carbon surface gradually decreases, leading to a significant reduction in the adsorption rate. Ultimately, PFAS molecules are slowly adsorbed onto the carbon surface until adsorption equilibrium is reached. Notably, in the partial dependence plot (Fig. 7c), a slight increase in adsorption capacity beyond 45 h is observed. The gradual increase in adsorption beyond 45 h is attributed to the slow diffusion of PFAS molecules into micropores, as well as the progressive exposure of new adsorption sites or the reorganization of surface functional groups, leading to a secondary uptake phase.^45^

The pH value has a significant effect on the removal efficiency of PFAS. As observed in the SHAP plot (Fig. 6b), increasing pH exerts a negative impact on adsorption. This trend is consistent with the partial dependence plot (Fig. 7d), which shows a gradual decrease in adsorption capacity with increasing pH. This behavior arises because pH influences the ionization state of PFAS molecules, the surface charge of activated carbon, and the speciation of other components in the solution. PFAS compounds contain negatively charged carboxyl or sulfonate functional groups. Under high pH conditions (alkaline), these groups are more likely to exist in their ionized forms, carrying negative charges, which increases their solubility in water and reduces adsorption onto carbon surfaces.^46^ This is due to electrostatic repulsion between the negatively charged PFAS molecules and the typically negatively charged activated carbon surface, resulting in diminished adsorption. Conversely, at lower pH values (acidic), PFAS molecules tend to remain in their unionized forms, reducing electrostatic repulsion with the carbon surface and enhancing adsorption. Studies have reported that at lower pH, numerous protons bind at the carbon–water interface, rendering the carbon surface positively charged, which strengthens electrostatic interactions with PFAS anions and promotes adsorption.^47,48^ As pH increases, the carbon–water interface becomes neutral or negatively charged,^49^ leading to electrostatic repulsion between the carbon surface and PFAS molecules, thereby decreasing adsorption capacity.

During the adsorption process, log *K*_ow_ significantly influences the adsorption performance. log *K*_ow_, the octanol–water partition coefficient, is a key parameter representing the distribution equilibrium of a compound between the aqueous and organic phases (commonly octanol), and is widely used to evaluate hydrophobicity and lipophilicity. Previous studies^30^ have shown that higher log *K*_ow_ values correspond to stronger hydrophobicity. Several studies reported that the adsorption capacity of carbon materials for PFAS increases with the hydrophobicity of PFAS.^30,50^ This indicates that PFAS with higher log *K*_ow_ values, reflecting stronger hydrophobicity, are more readily adsorbed onto carbon materials such as activated carbon, whereas PFAS with lower log *K*_ow_ values are more hydrophilic and thus more difficult to adsorb effectively. As observed from the SHAP plot (Fig. 6b), increasing log *K*_ow_ exerts a positive effect on adsorption, which is consistent with the trend observed in the partial dependence plot (Fig. 7e), where adsorption capacity increases with log *K*_ow_. These results indicate that as log *K*_ow_ increases, the hydrophobicity of PFAS molecules is enhanced, promoting interactions with the hydrophobic regions of the carbon surface and thereby increasing adsorption capacity. Both SHAP values and partial dependence plots confirm that log *K*_ow_ exerts a significant positive influence on adsorption, highlighting it as an important molecular feature affecting PFAS adsorption.

The effect of BET surface area on adsorption is discussed. In general, BET surface area plays a crucial role in adsorption, as a larger BET value typically provides more active sites, thereby leading to higher adsorption capacity.^41^ As shown in the partial dependence plot (Fig. 7f), the overall trend indicates that adsorption capacity increases with increasing BET surface area, demonstrating a positive correlation. Although the available data around 1000 m^2^ g^−1^ are scarce and the corresponding adsorption capacities remain relatively low, making it difficult to draw meaningful conclusions in this specific range, the overall trend is clear: adsorption capacity generally increases with the enlargement of BET surface area.

The carbon chain length and number of fluorine atoms in PFAS molecules are key factors affecting adsorption performance. As shown in the partial dependence plots (Fig. 7g and h), both carbon chain length (C) and fluorine atom count (F) exhibit a positive correlation with adsorption capacity. The results of this study indicate that both parameters play significant roles in adsorption. With increasing carbon chain length, adsorption capacity shows an upward trend, consistent with previous studies reporting that PFAS hydrophobicity increases with chain length.^51,52^ Longer carbon chains substantially enhance the hydrophobicity of the molecules, facilitating their separation from the aqueous phase and interaction with carbon-based adsorbents. Similarly, an increase in the number of fluorine atoms further strengthens molecular hydrophobicity and chemical stability,^53^ promoting enrichment on the carbon surface. Typically, a larger number of fluorine atoms confers stronger hydrophobicity to PFAS. The carbon chain length and the number of fluorine atoms, by modulating molecular hydrophobicity, molecular volume, and interactions with carbon-based adsorbents, significantly influence the removal efficiency of PFAS during adsorption processes. However, beyond a certain threshold of carbon chain length, further increases in fluorine content do not significantly enhance hydrophobicity, resulting in a plateau of adsorption capacity.^54^ Therefore, it can be inferred that within a certain range, the carbon chain length and fluorine atom count positively influence adsorption, while beyond this range, the enhancement effect on adsorption becomes negligible.

## 4. Conclusion

The performances of all models in predicting PFAS adsorption on carbon-based materials are summarized in Table 1. As shown, GBDT achieved the highest testing *R*^2^ with minimal error, indicating the best overall predictive performance among the models. The results indicate that the GBDT model achieved the best data fitting and predictive performance. Model interpretation revealed that the feature importance follows the order: initial concentration ratio of carbon-based material to PFAS > solution pH > reaction time > carbon specific surface area > log *K*_ow_ > number of fluorine atoms > carbon chain length > temperature.

The adsorption of PFAS is mainly governed by hydrophobicity, as indicated by positive correlations with log *K*_ow_, carbon chain length, and fluorine atom number; however, adsorption plateaus beyond a certain threshold. Solution pH affects electrostatic interactions, while the pore structure of carbon materials governs the ease with which PFAS molecules can access adsorption sites. The GBDT model accurately predicts PFAS adsorption under varying conditions and clarifies key influencing factors. Future studies should incorporate real environmental parameters and multi-PFAS systems to explore competitive and synergistic effects, enhancing understanding of carbon-based materials' efficiency for PFAS removal in practical applications.

## Author contributions

Yanliang Lu: conceptualization, methodology, data curation, formal analysis, validation, visualization, and writing – original draft. Fangfang Ding: investigation, data curation, and writing – review & editing. Guchun Wang: methodology, resources, and formal analysis. Yabin Li: data curation and visualization. Zhitao Guo: validation and investigation. Peiyao Pang: resources and investigation. Baojun Wang: supervision, project administration, funding acquisition, and writing – review & editing. Jue Liu: supervision, conceptualization, project administration, funding acquisition, and writing – review & editing.

## Conflicts of interest

The authors declare no competing interests.

## Supplementary Material

RA-015-D5RA07898A-s001

## Data Availability

The data as well as the code written for this study have been made publicly available on the GitHub link. The detailed calculation and programming procedure are made available *via* the following GitHub link (https://github.com/luyanliang-cat/PFAS-data/blame/main/data.zip). Supplementary information (SI): the datasets used in this study and the Python code for data preprocessing, model training, and analysis. See DOI: https://doi.org/10.1039/d5ra07898a.
