# Supplementary material for: Machine learning-based prediction and mechanistic insight into PFAS adsorption on carbon-based materials
Source: RSC Adv. 2025 Dec 8;15(56):48450–62. doi: 10.1039/d5ra07898a (PMC12684052; doi:10.1039/d5ra07898a)
Supplement: RA-015-D5RA07898A-s001 [file RA-015-D5RA07898A-s001.pdf]

Supplementary Information (SI) for RSC Advances.
This journal is © The Royal Society of Chemistry 2025

Supplementary data

Machine learning-based prediction and mechanistic insight into PFAS adsorption on carbon-based materials

Yanliang Lu[a], Fangfang Ding[a], Guchun Wang[a], Yabin Li[a], Zhitao Guo[a], Peiyao Pang[a], Baojun Wang[a*], Jue Liu[b*]

[a]National&Local Joint Engineering Research Center of Metrology Instrument and System, College of Quality and Technical Supervision, Hebei University, Baoding 071002, China

[b]Key Laboratory of Tropical Island Land Surface Processes and Environmental Changes of Hainan Province, School of Geography and Environmental Sciences, Hainan Normal University, Haikou 571158, China

* Corresponding author email: wbj498@163.com; jueliu@buaa.edu.cn;

1. Text

Text S1. Grid Search

Grid search in Python is employed to optimize GBDT hyperparameters by selecting the configuration that yields the lowest RMSE on the validation dataset. In the scikit-learn library, GBDT is implemented through the Gradient Boosting Classifier and Gradient Boosting Regressor.[1] The hyperparameters mainly involve boosting iterations and decision tree settings. In this study, the evaluated parameters were: n_estimators (100, 200, 300, 500, 1000), max_depth (3, 5, 8, 15, 20, 25, 30, None), min_samples_leaf (1, 2, 5, 10), learning_rate (0.01, 0.05, 0.1, 0.2), and max_features ("log2", "sqrt", None). The dataset was randomly divided into training and testing subsets with a ratio of 0.8:0.2 during the search process.

Text S2. SHAP Values

For each observation, the model outputs a predicted value, while SHAP assigns an importance score to every feature associated with that observation. Formally, SHAP values are computed by evaluating the marginal contribution of each feature through the difference in model predictions with and without the feature, followed by averaging across all possible feature coalitions. This process yields the Shapley value of the feature,[2] as defined in Eq. (S17).

$$Shapley\ value = \sum_{s \subseteq S_i} [(|s| - 1)!(n - |s|)!/n!][v(s \cup \{i\}) - v(s)] \tag{S17}$$

Where S_i denotes all subsets of features excluding feature i, $|s|$ is the cardinality of subset s, $v(s)$ represents the model prediction based on features in s, and $v(s \cup \{i\})$

corresponds to the model prediction including feature i.

Text S3. The partial dependence

The partial dependence function for regression is defined as shown in Eq. (S18):[3]

$$\hat{f}_{x_S}(x_S) = E_{z_C}[\hat{f}(x_S,x_C)] = \int \hat{f}(x_S,x_C)dP(x_C) \tag{S18}$$

Here, $\hat{f}_{x_S}(x_S)$ denotes the partial dependence of the response variable on the feature subset x_S, while $E_{z_C}[\hat{f}(x_S,x_C)]$ represents the expected value of the predicted outcome over the distribution of the remaining features x_C.The integral $\int \hat{f}(x_S,x_C)dP(x_C)$ calculates this expectation across the probability distribution $P(x_C)$. x_S is a subset of features used in the regression model, and x_C is its complement, i.e., the remaining features not included in x_S; $dP(x_C)$ denotes the probability distribution of x_C.

Text S4. Principal Component Analysis

Purpose:

PCA was applied to reduce the dimensionality of correlated variables (e.g., carbon and fluorine contents) while retaining the maximum variance.

Principle:

PCA transforms the original correlated variables $X = [x_1,x_2,...,x_p]$ into a new set of uncorrelated variables (principal components) $Z = [z_1,z_2,...,z_p]$by a linear combinatio:[4]

$$z_i = a_{i1}x_1 + a_{i2}x_2 + \cdots + a_{ip}x_p \tag{S19}$$

Where a_{ij} are the coefficients (loadings) obtained from the eigenvectors of the covariance matrix of X. The first principal component z_1 captures the maximum

variance of the original variables.

Usage:

In this study, the first principal component of the carbon chain length (C) and the number of fluorine atoms(F) was used as a new variable to replace the original C and F features in subsequent analyses.

Text S5. Datase

The dataset in this study was derived from 37 publications, resulting in a total of 605 data entries included in the final dataset. [5–41].

Figures:

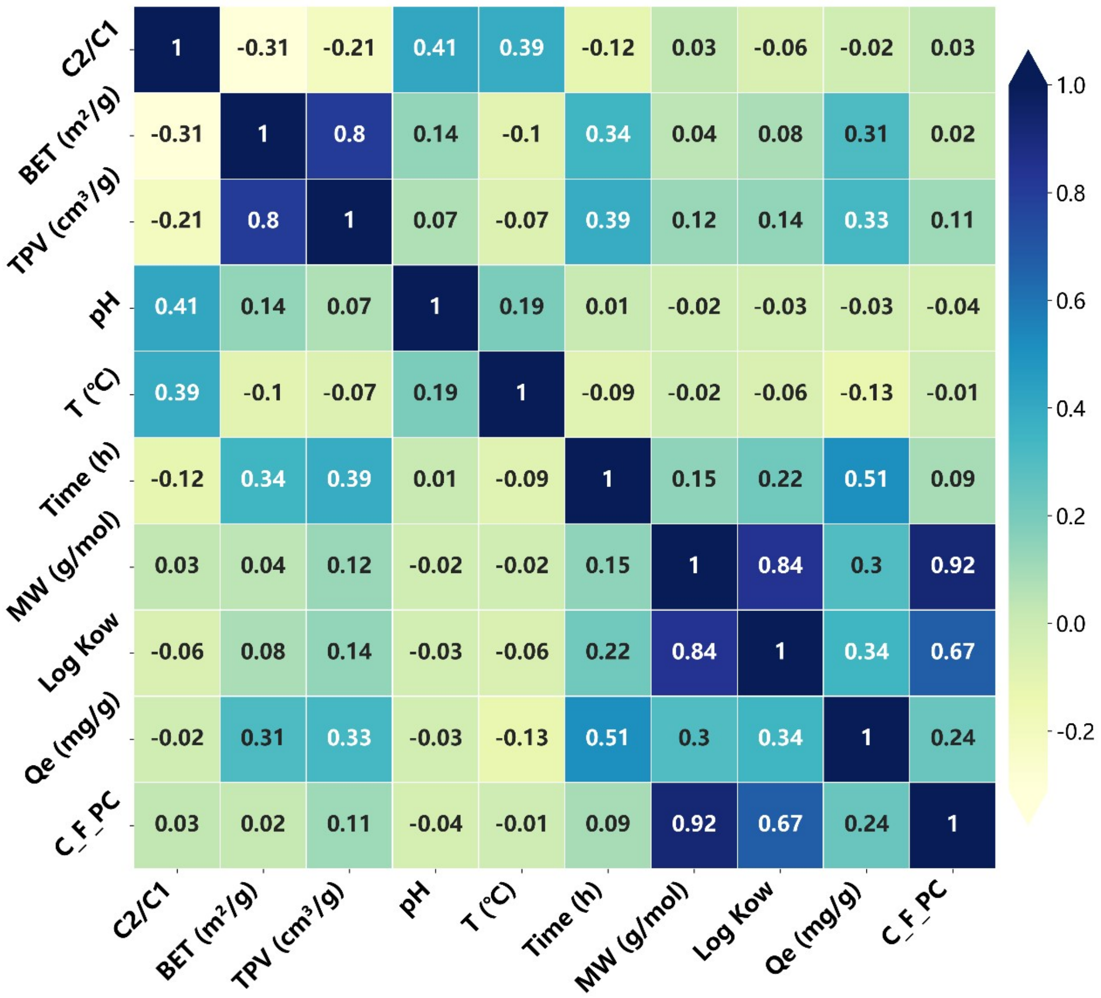

Fig. S1. Pearson correlation coefficient of input features.

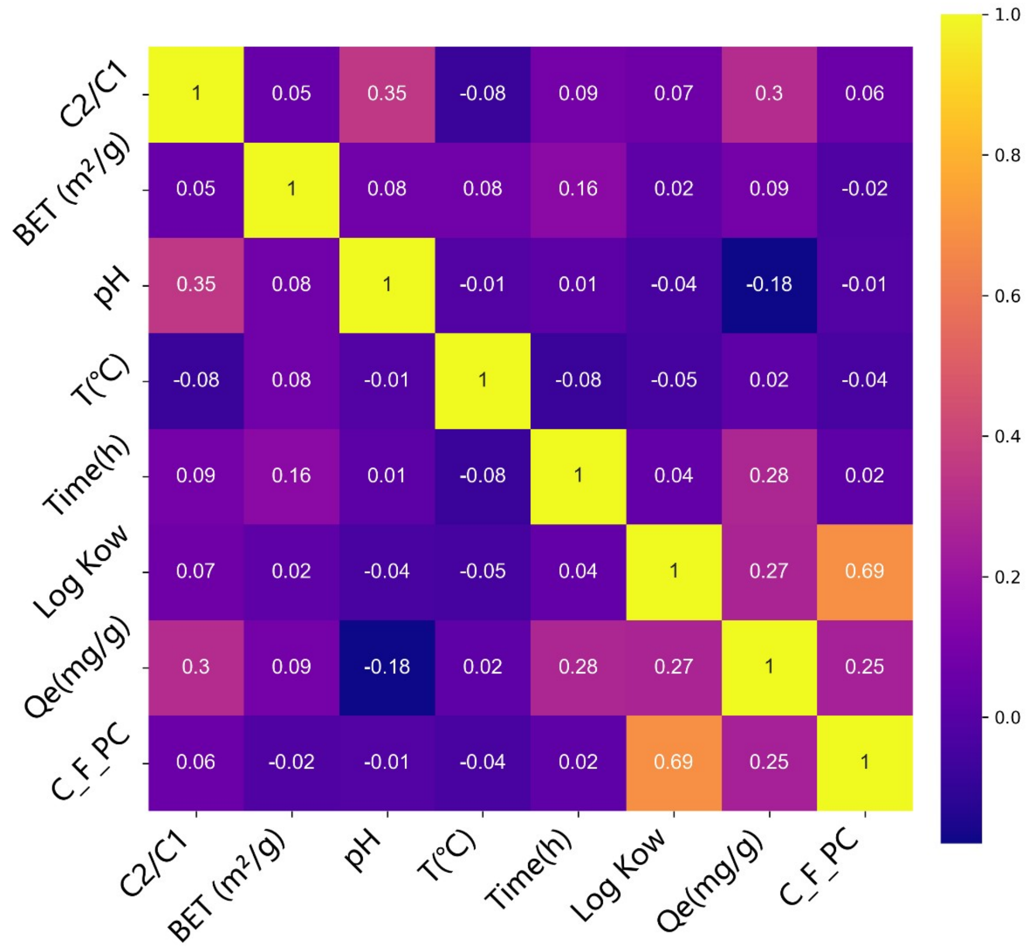

Fig. S2. Kendall correlation coefficient of input features.

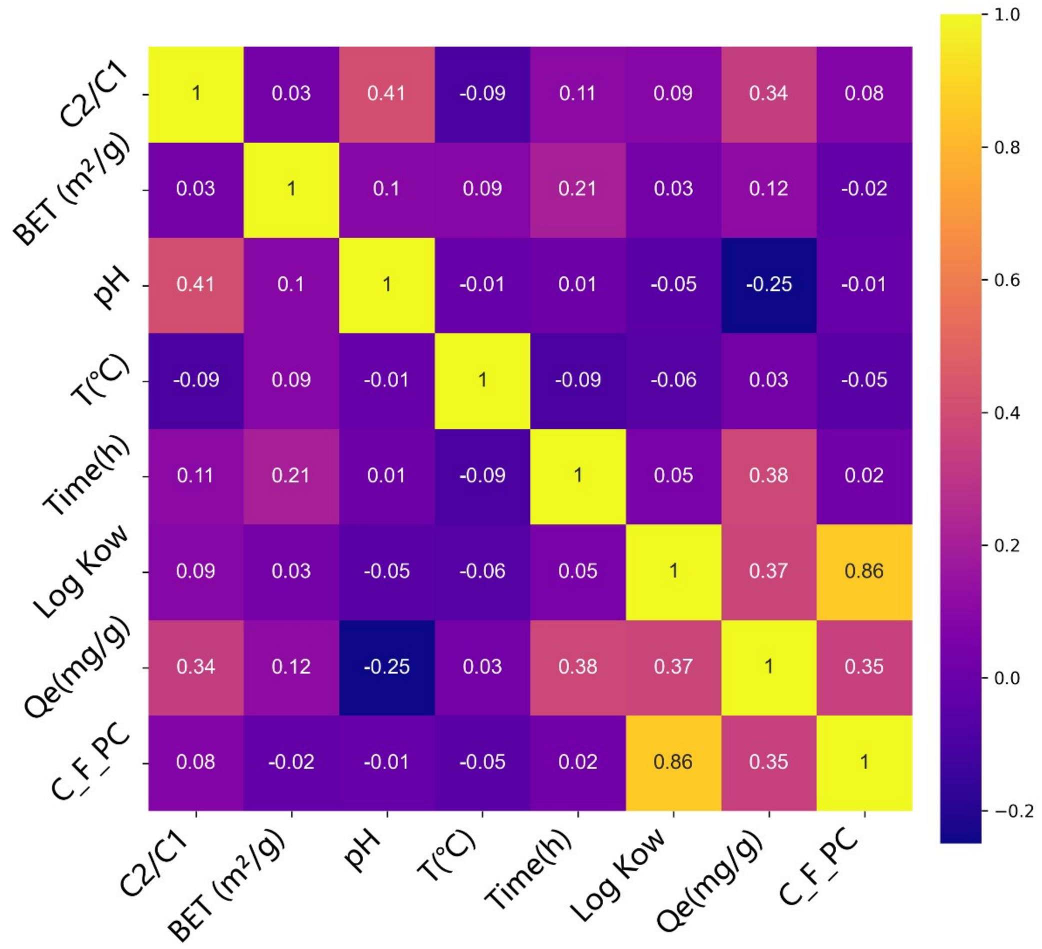

Fig. S3. Spearman correlation coefficient of input features.

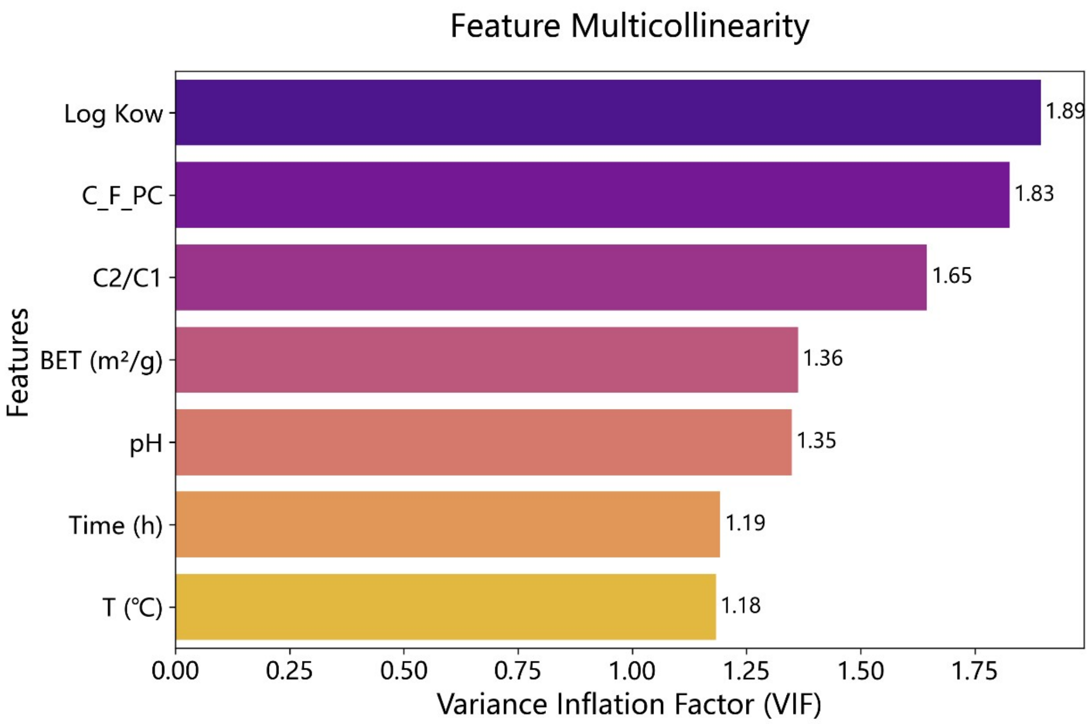

Fig. S4. Variance inflation factor (VIF) of input features.

References:

[1] D.A. Singer, *Math. Geosci.*, 2020, **53**, 675-687.

[2] E. Sánchez-Rodríguez, M.Á. Mirás Calvo, C. Quinteiro Sandomingo, I. Núñez Lugilde, *Int. J. Game Theory.*, 2023, **53**, 547-577.

[3] P. Bhandari and T. G. Lee, *J. Appl. Genet.*, 2024, **65**, 283–286.

[4] Yadav A K, Malik H, Chandel S S, *Renewable and Sustainable Energy Reviews*, 2015, **52**, 1093-1106.

[5] Saeidi N, Kopinke F D, Georgi A, *Chemical Engineering Journal*, 2020, **381**, 122689.

[6] Tan H M, Pan C G, Yin C, et al, *Environmental Research*, 2023, **233**, 116495.

[7] Inyang M, Dickenson E R V, *Chemosphere*, 2017, **184**, 168-175.

[8] Son H, Kim T, Yoom H S, et al., *Water*, 2020, **12(11)**, 3287.

[20] Pala J, Le T, Kasula M, et al, *Separation and Purification Technology*, 2023, **309**, 123025.

[9] Saawarn B, Mahanty B, Hait S, et al., *Environmental Research*, 2022, **214**, 114004.

[10] Sun R, Sasi P C, Alinezhad A, et al., *Journal of Hazardous Materials Advances*, 2023, **10**, 100311.

[11] Zhang D, Luo Q, Gao B, et al., *Chemosphere*, 2016, **144**, 2336-2342.

[12] Chen W, Zhang X, Mamadiev M, et al., *RSC advances*, 2017, **7(2)**, 927-938.

[13] Yu Q, Zhang R, Deng S, et al., *Water research*, 2009, **43(4)**, 1150-1158.

[14] Zhang D, He Q, Wang M, et al., *Environmental technology*, 2021, **42(12)**, 1798-1809.

[15] Mohamed B A, Li L Y, Hamid H, et al., *Chemosphere*, 2022, **294**, 133707.

[16] Salawu O A, Han Z, Adeleye A S, *Journal of hazardous materials*, 2022, **437**, 129266.

[17] Militao I M, Roddick F A, Bergamasco R, et al., *Journal of Environmental Chemical Engineering*, 2021, **9(4)**, 105271.

[18] Gagliano E, Sgroi M, Falciglia P P, et al., *Water research*, 2020, **171**, 115381.

[19] Fagbayigbo B O, Opeolu B O, Fatoki O S, et al., *Environmental Science and Pollution Research*, 2017, **24(14)**, 13107-13120.

[20] N. Gevaerd de Souza, A. C. Parenky, H. H. Nguyen et al., *Water Environ. Res.*, **2022**, 94, 1671.

[21] Ochoa-Herrera V, Sierra-Alvarez R, *Chemosphere*, 2008, **72**, 1588-1593.

[22] Du Z, Deng S, Chen Y, et al., *Journal of hazardous materials*, 2015, **286**, 136-143.

[23] Militao I M, Roddick F, Fan L, et al., *Journal of Water Process Engineering*, 2023, **53**, 103616.

[24] Chen R, Huang X, Li G, et al., *Science of The Total Environment*, 2022, **848**, 157723.

[25] Cantoni B, Turolla A, Wellmitz J, et al., *Science of The Total Environment*, 2021, **795**, 148821.

[26] Umeh A C, Hassan M, Egbuatu M, et al., *Science of the Total Environment*, 2023, **904**, 166568.

[27] Krebsbach S, He J, Adhikari S, et al., *Chemosphere*, 2023, **330**, 138661.

[28] H. Son and B. An, *Water Air Soil Pollut.*, 2022, **233**, 129.

[29] D. M. Kempisty, E. Arevalo, A. M. Spinelli et al., *AWWA Water Sci*., 2022, **4**, 1269,

[30] J. Fabregat-Palau, M. Vidal and A. Rigol, *Chemosphere*, 2022, **302**, 134733.

[31] Y. Qu, C. Zhang, F. Li, et al., *J. Hazard. Mater.*, 2009, **169**, 146–152.

[32] S. Deng, Y. Nie, Z. Du, et al., *J. Hazard. Mater.*, 2015, **282**, 150–157.

[33] N. Liu, C. Wu, G. Lyu, et al., *Sci. Total Environ.*, 2021, **798**, 149191.

[34] Y. Yao, K. Volchek, C. E. Brown, et al., *Water Sci. Technol.*, 2014, **70**, 1983–1991.

[35] Y. Zhang, A. Thomas, O. Apul, et al., *J. Hazard. Mater.*, 2023, **460**, 132378.

[36] W. Guo, S. Huo, J. Feng, et al., *J. Taiwan Inst. Chem. Eng.*, 2017, **78**, 265–271.

[37] D. Q. Zhang, W. L. Zhang, Y. N. Liang, *Sci. Total Environ.*, 2019, **694**, 133606.

[38] Y. Zhi, J. Liu, *Environmental pollution.*, 2015, **202**, 168-176.

[39] J. M. Steigerwald, J. R. Ray, *Journal of Hazardous Materials Letters.*, 2021, **2**, 100025.

[40] X. Chen, X. Xia, X. Wang, et al., *Chemosphere*, 2011, **83**, 1313–1319.

[41] A. C. Umeh, M. Hassan, M. Egbuatu, et al., *Sci. Total Environ.*, 2023, **904**, 166568.
